# Cetirizine a histamine H1 receptor antagonist improves viral myocarditis

**DOI:** 10.1186/1476-9255-7-39

**Published:** 2010-08-04

**Authors:** Akira Matsumori, Kanjo Yamamoto, Miho Shimada

**Affiliations:** 1 Department of Cardiovascular Medicine Kyoto University Graduate School of Medicine, Kyoto, Japan

## Abstract

**Background:**

We showed that mast cells played a critical role in the progression of heart failure induced by pressure overload and viral myocarditis in mice. In this study, we investigated the effect of cetirizine, a selective H1 receptor antagonist, on experimental viral myocarditis induced by encephalomyocarditis (EMC) virus.

**Methods:**

Four-week-old inbred male DBA/2 mice were inoculated intraperitoneally with 10 plaque-forming units (pfu) of the EMC virus. Cetirizine was administered orally at a dose of 1 or 10 mg/kg per day for the survival study, and 1 mg/kg for the histologic and gene expression studies, beginning on the day of viral inoculation.

**Results:**

Cetirizine improved survival dose dependently. Heart weight to body weight ratio was significantly decreased in mice treated with cetirizine. The area of myocardial necrosis was significantly smaller in the hearts of mice treated with cetirizine compared with controls. Gene expressions of tumor necrosis factor, interleukin 6, and metalloproteinase 2 were significantly suppressed in the hearts of mice treated with cetirizine.

**Conclusion:**

These results suggest that cetirizine exerts its beneficial effects on viral myocarditis by suppressing expression of pro-inflammatory cytokines, genes related to cardiac remodeling in the hearts of mice.

## Introduction

In recent years, mast cells have been implicated in the pathogenesis of cardiovascular and atherosclerotic disorders. In particular, we have observed that mast cells cause apoptosis of cardiac myocytes and proliferation of nonmyocytes in vitro [1]. Furthermore, myocardial histamine and tryptase content, and mast cell density are higher in heart failure due to idiopathic dilated or ischemic cardiomyopathy than in control hearts [2]. We showed that mast cells played a critical role in the progression of heart failure induced by pressure overload and viral myocarditis in mice [3,4]. In our previous study, mast cell deficient mice developed less pronounced myocardial necrosis and cellular infiltration induced by encephalomyocarditis virus, and the histamine H1-receptor antagonist improved survival of mice and in improved histological changes [4].

In the present study, we studied the effects of a histamine H1-receptor antagonist, cetirizine on the expressions of inflammatory cytokines and metalloproteinases on experimental viral myocarditis induced by encephalomyocarditis (EMC) virus which play important roles in cardiac remodeling.

## Methods

### Experimental myocarditis model

Stocks of the myocardiotrophic variant of EMC virus were prepared as previously described [5,6], and stored at -80°C. The 4-week-old male DBA/2 mice used in this study were treated in accordance with local institutional guidelines at all stages of the experiments. A total of 50 mice were inoculated with 0.2 ml EMC virus in phosphate buffered saline diluted to a concentration of 10 pfu/ml on day 0. The histamine H1-receptor antagonist cetirizine was purchased from Sigma (Tokyo, Japan). Cetirizine was dissolved in distilled water and given orally by gavage at a dose of 1 or 10 mg/kg per day starting on the same day on 1 or 10 mg/kg per day starting on the third day as the EMC virus inoculation (each n = 10). Control mice were given distilled water. For the histologic study, and the gene expression study, the study groups were control (n = 10), and cetirizine 1 mg/kg (n = 10). Control mice were given 0.2 ml of distilled water. At day 5, we observed that some mice began to die, which was expected, and could have been due to viremia and/or encephalitis. Surviving mice were sacrificed by cervical dislocation at day 5 for the gene expression study and at day 6 for the histopathologic experiments. The hearts were dissected, immediately frozen and stored at -80°C, and the section of interest fixed in formalin.

### Heart Weight and Lung Weight

Heart, lung and body weight were measured and the heart and lung weight/body weight was calculated.

### Histopathological examination

We examined the histopathologic changes on day 6. The hearts were fixed in 10% formalin, and embedded in paraffin. The left ventricles (LV) were sliced horizontally to the long axis, and stained with hematoxylin - eosin, and Masson's trichrome for light microscopy examinations. The extent of myocardial necrosis was evaluated by measuring the ratio (%): myocardial necrosis area/total LV area on a microscopic slide, using Microanalysis (Ather Coporation, Tokyo, Japan), which can measure areas of different colors. We calculated the area of myocardial necrosis, as indicated by the loss of red Masson's trichrome stain. Two investigators determined the histologic score, which were averaged. The analyses were blinded. To determine the number of mast cells, the hearts were stained with toluidine blue. The total number of mast cells in a given section (whole heart) was calculated as cells/mm^2^.

### Assay of myocardial virus concentration

Ten mice from each of the cetirizine or control group were sacrificed 6 days after EMCV virus inoculation. Myocardial viral concentrations were determined only in the sacrificed mice using an FL (human amnion cells)-plaque assay and expressed as pfu/mg of myocardium as described previously [7].

### Quantitative reverse transcriptase polymerase chain reaction analysis

A total of 18 of the 20 mice were studied for gene expression. One mouse of the cetirizine 10 mg/kg group and one control mouse died before day 5 and thus were not appropriate for study because of post-mortem changes.

Total RNA was isolated from the LV using the acid guanidinium thiocyanate-phenol-chloroform method and the RNA concentration was measured spectrophotochemically. First-strand cDNA was synthesized using SUPERSCRIPT Preamplification System for First-Strand cDNA Synthesis (GIBCO BRL). Real-time quantitative PCR (TaqMan PCR) using an ABI PRISM 7700 Sequence Detection System and TaqMan PCR Core Reagent Kit (Perkin-Elmer Corp, Foster City, CA) was performed according to the manufacturer's protocol. We used 2 μl of the First-strand cDNA, and the following forward (F) and reverse (R) oligonucleotides, and probes (P) were used for the quantification of tumor necrosis factor (TNF) α, interleukin (IL)-6, and matrix metalloproteinases (MMPs) 2and 9.

TNF-α F, 5'-CATCTTCTCAAAATTCGAGTGACAA;

TNF-α R, 5'-TGGGAGTAGACAAGGTACAACCC;

TNF-α P, 5'-CACGTCGTAGCAAACCACCAAGTGGA;

IL-6F, Based on TaqMan produt No.4331348

IL-6R, Based on TaqMan produt No.4331348

IL-6P, Based on TaqMan produt No.4331348

Inducible Nitric Oxide Synthase (iNOS)

iNOSF, 5'- CAGCTGGGCTGTACAAACCTT-3'

iNOSR, 5'-CATTGGAAGTGAAGCGTTTCG-3'

iNOSP, 5'-CGGGCAGCCTGTGAGACCTTTGA-3'

MMP-2 F, 5'-ACTGACCTGCATGGAATCAGC-3'

MMP-2 R, 5'-GGTTACTTGAGTGTTCTAGCCCA-3'

MMP-2 P, 5'-TCTTTCTGGTGGCCGTGCATGA-3'

MMP-9 F, 5'-TTGTGGTCTTCCCCAAAGACC-3'

MMP-9 R, 5'-TATCCACCGAGCCATCTGTCTA-3'

MMP-9 P, 5'-AAAACCTCCAACCTCACGGACACCCA-3'

GAPDH F, 5'-TTCACCACCATGGAGAAGGC-3';

GAPDH R, 5'-GGCATGGACTGTGGTCATGA-3';

GAPDH P, 5'-TGCATCCTGCACCACCAACTGCTTAG-3'.

The conditions for the TaqMan PCR were: 95°C for 10 min, followed by 40 cycles at 95°C for 15 s and 60°C for 1 min.

### Statistical analysis

The survival rate of mice was analyzed by the Kaplan-Meier method, and survival differences between groups were tested by the log-rank test. Statistical comparisons of histological area and gene expressions were made by the unpaired 2-tailed Student *t *test. All values are presented as mean ± SD. Differences were considered statistically significant at probability values < 0.05.

## Results

### Survival

**Figure 1:**
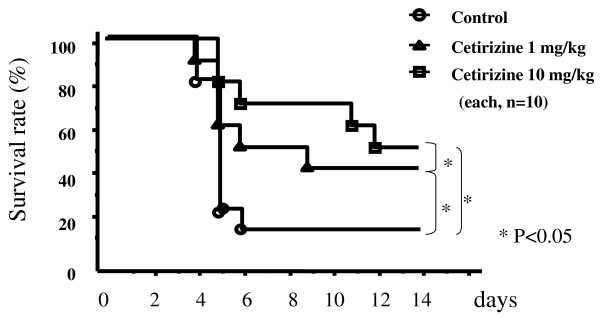
**Cetirizine improved survival dose dependently in mice treated with 1 or 10 mg/kg compared with controls (p < 0.05)**.

Cetirizine improved survival dose dependently in mice treated with 1 or 10 mg/kg compared with controls (p < 0.05, Figure 1). However, there was no significant difference in survival when cetirizine was started on the third day (survived mice in cetirizine treated mice, n = 2 vs in control, n = 1).

### Heart weight and lung weight

**Figure 2:**
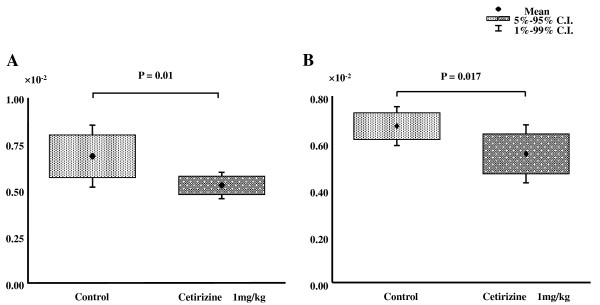
**Effect of cetirizine on heart weight/body weight ratio (A), and lung weight/body weight (B) ratios in EMC viral myocarditis in mice**.

Heart weight to body weight ratio was significantly decreased in mice treated with cetirizine ([0.53 ± 0.06] × 10^-2^, n = 9, mean ± SD) compared with controls ([0.68 ± 0.15] × 10^-2^, n = 9, p = 0.01, Figure 2A). Lung weight to body weight ratio was significant lower in mice treated with cetirizine ([0.55 ± 0.12] × 10^-2^, n = 9) compared with controls ([0.67 ± 0.074] × 10^-2^, n = 9, p = 0.02, Figure 2B).

### Myocardial Histology

**Figure 3:**
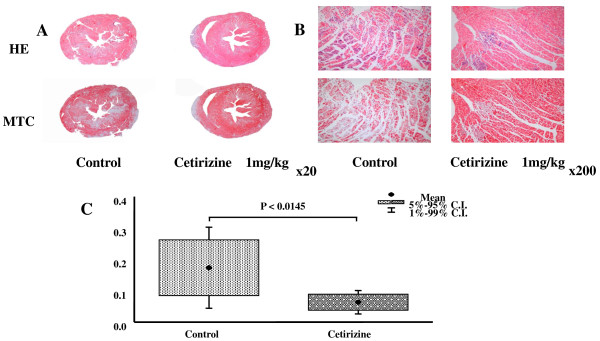
**Effect of cetirizine on histopathology of mice hearts with EMC viral myocarditis**. Representative pictures of the heart of mice treated with 1 mg/kg of cetirizine and control mice. A. × 20. B. × 200. HE: Hematoxylin-eosin stain. MTC: Masson's trichrome stain. C. Quantitation of myocardial necrosis.

The area of myocardial necrosis on day 6 was significantly less severe in the hearts of mice treated with cetirizine 1 mg/kg (6.3 ± 0.3%) compared with controls (17.3 ± 11.6%, p = 0.02) (Figure 3C). The area of necrosis was not improved when cetirizine was started on day 3 (15.6 ± 12.3, n = 8 vs Control 12.9 ± 4.4, n = 7, p = 0.59). Mast cell density did not show significant difference between cetirizine 1 mg/kg and control group (0.80 ± 0.40, n = 9) vs (0.95 ± 0.64, n = 9, p = 0.6).

### Myocardial viral concentration

Myocardial virus concentration on day 7 was (0.11 ± 0.02pfu/mg) in cetirizine treatment mice (n = 10) and (0.13 ± 0.01pfu/mg, n = 10) in control mice (n = 10). There was significant difference between the two groups (p < 0.05).

### Gene Expressions

**Figure 4:**
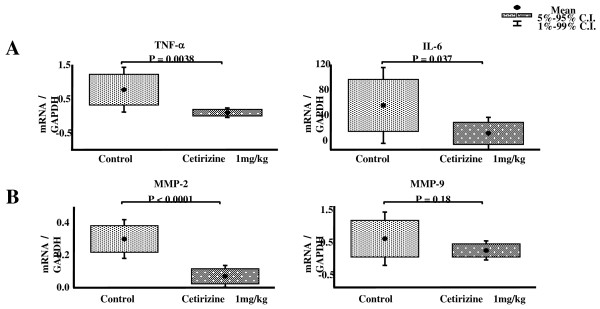
**Effect of cetirizine on gene expressions of TNF-α and IL-6 (A), and MMP-2 and MMP-9 (B) in the heart of mice with EMC viral myocarditis**.

The gene expressions of TNF-α and IL-6, inflammatory cytokines were significantly decreased compared with controls (TNFα/GAPDH: 0.09 ± 0.12 vs 0.77 ± 0.59, p = 0.0038; IL-6/GAPDH: 12 ± 23 vs 56 ± 53, p = 0.0371; each n = 9) in the hearts of mice treated with cetirizine 1 mg/kg (Figure 4A).

The gene expressions of MMP-2, a key molecule in cardiac remodeling, was significantly lower in the hearts of mice treated with cetirizine compared with controls (MMP-2/GAPDH: 0.07 ± 0.06 vs 0.3 ± 0.1, p < 0.0001; Figure 4B). A trend for a reduction in MMP-9 was seen (MMP/GAPDH: 0.14 ± 0.26 vs 0.5 ± 0.73, p = 0.19) in the cetirizine group that did not reach statistical significance.

The gene expressions of iNOS tended to be lower in the cetirizine than in the control group, but the differences did not reach statistical significance (iNOS/GAPDH: 0.114 ± 0.118 vs 0.920 ± 1.253, p = 0.073).

## Discussion

In our model of EMC virus myocarditis, the number of mast cells was increased on day 14 after EMC virus inoculation, when myocardial fibrosis becomes apparent [8], and in W/W^v ^and SI/Si^d ^strains of mice, we observed that mast cell deficiency had beneficial effects in the disorder.

We have reported that the gene expression of mast cell chymase and tryptase was upregulated in the acute phase of viral myocarditis and rose further in the subacute phase of heart failure [8]. This activation coincided with the development of myocardial necrosis and correlated with the upregulation of MMP-9 and type-I procollagen, suggesting that mast cell chymase and tryptase participate in the acute inflammatory reaction as well as the remodeling process associated with acute viral myocarditis.

Evidence is growing that pro-inflammatory cytokines play an important role in modulating cardiovascular function and structure [9-11]. Arteriovenous IL-6 spillover in the peripheral circulation increases with the severity of heart failure, and an elevated level of plasma IL-6 was a predictor of mortality in patients with heart failure [12].

In the present study, cetirizine improved survival of mice, congestion of the lungs, and myocardial necrosis, suppressed the expression of a pro-inflammatory cytokines and decreased expression of MMP-2. Thus, these may be the mechanisms by which cetirizine decreases inflammation and fibrosis. The results suggest that histamine released from mast cells may play a pivotal role in the pathogenesis of viral myocarditis. However, antihistaminic agents, such as cetirizine, not only act via mediation of H1 receptors, but may also attenuate various steps in the inflammatory process. A delay of three days after viral inoculation vastly reduced its efficacy in reducing adverse responses to viral myocarditis. Therefore, cetirizine should be started as early as possible in treatment of viral myocarditis.

Cetirizine has demonstrated several modulatory effects on inflammatory responses. These effects included reducing eosinophil migration induced by inflammatory mediators in atopic and nonatopic adults, reducing the expression of adhesion molecules associated with eosinophil migration and adhesion of eosinophils to epithelial cells and inhibiting the expression of various pro-inflammatory cytokines and mediators *in vitro *and *in vivo *[13].

A histamine H2 receptor block has been shown to be beneficial in human heart failure [14]. We have shown that mast cells stabilizer tranilast prevented development of heart failure in an animal model of pressure overload [3]. Therefore, not only H1 receptor blockers, but also other agents which stabilize mast cells may have beneficial effects on heart failure.

Although the exact molecular mechanisms of the beneficial effect of cetirizine remains to be clarified, cetirizine is a promising agent for the treatment of viral myocarditis and merits further study.

## Authors' contributions

AM designed the study, performed statistical analysis, and drafted the manuscript. KY and MS carried out animal experiments, performed histological and molecular studies. All authors read and approved the final manuscript.
